# Medical Opinion and Movement

**Published:** 1910-07-02

**Authors:** 


### Medical Opinion and Movement.

### Magnesia and Attacks of Angina.

It is well known that in patients liable to attacks of angina pectoris, of the so-called false kind and even of true angina, gastric conditions frequently afford an exciting cause for an attack, and a spontaneous evacuation of gas from the stomach often produces a marked relief and a diminution in the acute symptoms. In view of these facts Dr. V. von Chlapowski, of Kissingen, proposes to treat such patients by administering substances having the power to absorb gases, and for this purpose he thinks the most suitable is magnesia—either calcined magnesia or magnesia hydrate. He gives it in doses of 50 centigrammes after each meal or as soon as the patient begins to feel the first symptoms of oppression or pain. He is convinced that the magnesia is successful in diminishing the intensity of the attacks not only in cases of false angina but also when there is some definite cardiac lesion. As he points out, even in the latter cases the attacks of angina not infrequently have their causal origin in some disturbance of the digestive function—acid dyspepsia, meteorism, intestinal fermentation, and the like. The treatment is at least simple and quite harmless, and the magnesia can be administered over a prolonged period without producing any intolerance to the drug or other inconvenience.

### Human Blood for Pernicious Anæmia.

It has been shown that intravenous injections of defibrinated human blood have exercised a favourable effect upon the course of the disease in certain cases of pernicious anæmia, but unfortunately in spite of all precautions it is difficult to avoid certain evil results from these blood transfusions, such as sudden collapse, acute dyspnœa with cyanosis, rigors with fever, and so on. It seems improbable, however, that the increase observed in the hæmoglobin content and number of red corpuscles is directly due to the transfused blood, but the favourable result is rather explained by an anti-toxic action or by an excitation of the hæmatopoetic function of the bone-marrow. The question arises, therefore, whether a like effect might not be obtained by recourse to intramuscular injections. Dr. O. Huber, of the Augusta Victoria Hospital at Schöneberg, has acted on this view, and his results appear to fully justify the supposition. He made the injections into the muscles of the buttocks, beginning with 10 cubic centimetres of defibrinated blood, and increasing the quantity sometimes to as much as 50 cubic centimetres. They are repeated at intervals of five to eight days. They are tolerated rather differently by different patients, in some even small doses producing painful swellings with a little rise of temperature, while in others the dose can easily be increased up to 40 or 50 c.c. Clinical details of several cases are given, in which the injections were rapidly followed by an increase of the hæmoglobin from 20 or 40 to 75 or 90 per cent., and the red corpuscles from one or two to four or five millions. At the same time the body weight increased by 5 or 6 kilos. These results were generally obtained in the course of two to three months. The author does not claim that these injections will effect a cure in every case. In some they have failed to do more than effect a temporary amelioration, but if the disease is treated before it is too far advanced, and before the internal organs have undergone serious changes, it seems possible successfully to arrest it by these means.

### High-frequency Currents for Diabetes.

Dr. J. M. Liebermann, of New York, has carried out the treatment of glycosuria by means of high-frequency currents in large numbers of cases, and has obtained success in a considerable proportion. He supposes that the glycosuria is connected with a hyperæmia of the blood-vessels to the liver, and he attempts to counteract this hyperæmia by acting upon the sympathetic system with the electric current. He makes the applications, therefore, in the epigastric region immediately over the cœliac plexus, semilunar ganglia and splanchnics, directing the current from left to right. The applications are made for 10 to 15 minutes at sufficient intervals to prevent exhaustion. As a result of these applications the patient has a feeling of wellbeing, and the chief symptoms of the disease—polyuria, thirst, and polyphagia—soon commence to diminish. At the same time there is a decrease in the amount of sugar excreted, and the general condition of the patient improves. The duration of the treatment should extend over six months to a year. Of 144 cases so treated he has had 62 relative failures, which he ascribes in a large measure to insufficient treatment. Even in these 62 cases there was almost always a diminution in the glycosuria, and an improvement in the general condition and an increase in body weight.

### The Ultimate Fate of Choreics.

In the *Jahrbuch für Kinderheilkunde* there appears an interesting report by Dr. G. Forssner on the ultimate fate of patients who were treated at the clinic of the Stockholm University from 1885 to 1892 for cardiac affections, articular rheumatism, and chorea. Of thirty-four cases of chorea he was able to follow up twenty-eight, and of these seven had died subsequently from heart trouble, five were afflicted with a more or less severe cardiac affection, five patients developed pulmonary phthisis, which had already proved fatal in two cases, and in two other cases there was also heart trouble. In seven cases there was albuminuria with more or less severe cardiac lesions, and in one of these cases there was also a goitre. In one other case there was a goitre with cardiac trouble, probably Basedow's disease. In another case the patient was suffering from gastric trouble with loss of flesh, and in yet another there was marked mental weakness. So that as a result of this inquiry it was found that of these twenty-eight cases, in only one did the condition of health appear to be normal. Five of the patients suffering from heart trouble had died before reaching eleven years of age, and of the remaining twenty-three at least fourteen had developed some chronic affection which could not be accounted for as a complication of chorea. The author is convinced that such chronic affections as tuberculosis, nephritis, and goître occur much less frequently among patients affected with some other disease than chorea during their childhood, and he concludes from this inquiry that chorea especially attacks those of a feeble constitution with little power of resistance to other diseases also.

### Herpes Zoster in Childhood.

In a recent *Thèse de Paris* Madame Galka describes the chief differences which exist between herpes zoster as it exists in the child, and the same disease as it is met with in the adult. Up to the age of two years the disease is very rarely seen, but girls would seem to be more frequently attacked than boys. In nearly every case gastric disturbances play an important part in the ætiology of the affection, contrary to what is the rule in adults. Also it is usually a benign disease in childhood, and lasts only from eight to ten days, instead of 20 to 30. The symptomatology is much the same at all ages, but the intensity of the disease and the duration of the pain is markedly less in children. Fever would also seem more frequently present in the adult. Intercostal zona is the most common form at all ages, but ophthalmic zona is rare in children, and when it occurs is much less painful and leaves less trace than it does in the adult. As a rule there is swelling of the glands draining the affected area, and pain and tenderness are sometimes present in them. The disease may be bilateral and show bifurcations in the child. The vesicles are at times filled with a sanious fluid, but herpes never becomes hæmorrhagic or gangrenous, as it does at times in the adult. The child also has the advantage in that zones of hyperæsthesia and anæsthesia never result from an attack of herpes zoster. Treatment is simple, consisting in mild purgation and the application of an inert powder to the vesicles. If the latter are excoriated they should be bathed with some weak demulcent fluid and kept exceedingly clean.

### Anæsthesia by Electricity.

Dr. Louise Robinovitch, in the *Journal of Mental Pathology*, describes a new method of inducing anæsthesia by means of the constant electric current. Zinc electrodes, covered with a thick layer of absorbent cotton wetted with salt solution, are used, the kathode, which should be large enough to cover the forehead, being placed on the head, and the anode on the lumbar region of the patient. A constant current, capable of interruption at the negative pole, and of a strength of five to ten volts, with a milli-amperage ranging between 1.5 and 2 m.a., will anæsthetise a dog, the resistance of which varies from 300 to 500 ohms, according to the size of the animal and of the electrodes used. At first the animal is uneasy, and as the strength of the current increases it becomes agitated and struggles. Eventually it falls on one side and remains quiet. If the anæsthetic is required for a long time, the voltage should now be gradually decreased. During such anæsthesia the animal's eyes remain open, and it occasionally lifts its head for a second or two, and may even attempt to liberate itself, as consciousness is not entirely lost. The cutaneous reflexes are increased, but the sense of touch and sensibility to pain are markedly decreased, though not abolished entirely. The dog, which is usually the most sensitive to pain of all animals used in laboratory work, stands well the most painful operations under this anæsthetic. At the hands of the author no untoward accidents have happened during an experience extending over three years. Among the sequelæ which follow electric anæsthesia are incontinence of fæces and urine, and abortion either on the same day or the day following the anæsthetic. If the animal be in milk, this is projected with force at the beginning of the electrisation. Otherwise there are no sequelæ, the animal walking about immediately after the operation. Local anæsthesia can be similarly produced by applying both the anode and the kathode along the sensory nerve. The author lays great stress on the necessity of a daily experience of at least two years' duration with laboratory animals before applying electric anæsthesia to the human subject. Moreover, the method should never be applied to persons who are past middle age or suffering from arterio-sclerosis, epilepsy, or apoplexy, since the blood-pressure is always raised by the electric stimulation. Again, on no account should electric anæsthesia be combined with any other anæsthetic, even morphia, since the subject is profoundly agitated if the electric stimulation is kept up during the administration of a second anæsthetic. The article closes with a complete account of the technique and instrumentation necessary to apply the method successfully.

### Resuscitation by Electricity.

In another part of the same journal the author discusses the methods at present in use for the resuscitation of subjects in a condition of apparent death as the result of inhalation of chloroform and ether, or of electrocution or of drowning. She then proceeds to describe a method discovered by herself in which respiratory movements of required amplitude and cardiac beats are produced by electric currents. To illustrate her method, she describes the manner in which a dog can be first poisoned with an overdose of chloroform and then resuscitated. After fixing the animal in a Claude Bernard cradle, care being taken to fix the head and paws, which may otherwise be injured during the period of excitement, the animal is anæsthetised by the electric method described above; the pneumograph is fitted to its chest, and the blood-pressure taken by the manometer at the carotid or femoral artery. When the animal is fully anæsthetised electrically, chloroform anæsthesia is substituted for the former method and rapidly pushed until an overdose has been taken. As soon as apparent death has taken place with arrested blood-pressure and respiration of air, preparations are made to revive the animal. Its back is shaven in two places, at the lumbar and dorsal regions respectively, to which are applied cotton-covered zinc electrodes soaked in saline solution. The anode is placed at the lumbar, the kathode at the dorsal region, care being taken to exclude the animal's head from the circuit. The front paws and head are then set free, and the tip of the tongue seized with forceps preparatory to clearing out the mouth and throat of saliva and mucus. Various electric currents should be at hand, such as alternating, constant, frequently interrupted, and current induced from a coil. The kathode is connected with the switch in the circuit of the current to be utilised, and the anode with the potential reducer, by means of which the voltage can be controlled. The animal's mouth being kept well open, the operator commences to practise rhythmic electric excitations with the current chosen—*e.g.* one of low tension and frequent interruption. The first excitations are produced with the minimum voltage necessary to cause maximum inspirations, generally from 15 to 20 volts. The first closure of the circuit, lasting from a quarter to half a second, causes energetic artificial respiration. In less grave cases the heart will react at once, but when syncope is profound, the voltage should be increased. As long as the blood-pressure remains constant there is no necessity to increase the current. After a time in suitable cases feeble spontaneous respirations will follow, as well as spontaneous heart-beats. In addition to cases of apparent death from chloroform and ether narcosis, the author has been able to revive a dog which to all appearance had died from a severe accidental hæmorrhage. She claims that, because of its ease in application, the means should be at hand of applying the method in all surgical operations in the human subject. In cases of drowning, non-obstructive angina pectoris, and apparent death from electrocution the method is especially applicable.

### Indian Ink and the Treponema Pallidum.

Gurd, in the *Journal* of the American Medical Association, May 28, 1910, gives a good description of the new method of demonstrating the *Treponema pallidum* by the use of ordinary Indian ink. The method depends on the fact that such ink does not stain bacteria or spirochætes, the organisms in the specimens remaining white, but showing up very distinctly against the brownish-black background. The technique of the method is extremely simple, and if eventually proved to be reliable by competent workers, will be the means of putting the microscopic diagnosis of syphilis before anyone possessing a high-power microscope. Regarding the reliability of the method Dr. Gurd says it is not yet possible to speak definitely. It would appear, however, from his own results that it is a method by which a definite diagnosis of syphilis is made possible in as great a number of cases as by any other microscopic method. He examined a series of cases using Giemsa's and Wright's stains as controls, and in every case in which either of these staining methods revealed the organisms the Indian-ink method demonstrated them equally well and with a much smaller expenditure of labour and time. More observations will be required to prove the efficacy of this method, but if found not to be wanting in accuracy, then naturally it will come to be generally used in the microscopical diagnosis of syphilis.

### The Comparative Anatomy of the Prostate.

That the *prostatic gland* is not altogether a uniform structure such as has hitherto been generally supposed seems to be indicated by some recent investigations carried out by Dr. George Walker at Johns Hopkins University and published in the *Bulletin* of the Johns Hopkins Hospital. Investigating the structure of the prostate of rats and guinea-pigs in particular, it was found that embedded in the same sheath as the vesiculæ seminales, and yet forming part of the prostatic gland, there are more structures than one, and he has found that the secretion of the prostatic gland proper and that of a glandular structure adjacent to the seminal vesicles are radically different in physical and chemical characteristics. In neither the rat nor the guinea-pig does the secretion of the prostate gland coagulate that of the seminal vesicles, but in both the rat and guinea-pig the secretion of the glandular structure adjacent to the seminal vesicles, which has been termed the "coagulating gland," coagulates the seminal fluid. This coagulation is produced by a very minute quantity of the secretion, 1 part of it to about 21,000 parts of the seminal vesicle secretion being sufficient to produce the reaction. The phenomenon seems to be entirely different from blood coagulation, though the active principle in the secretion of the coagulating gland presumably belongs to the class of ferments. The seminal vesicle secretion of the rat probably contains histone, whilst in the seminal vesicle secretion of the guinea-pig there is a substance which corresponds in part to histone, and in part to the higher proteins. The spermatozoa of the rat are immediately clumped when mixed with the seminal vesicle secretion of the same animal. This phenomenon does not occur in the guinea-pig, neither does the seminal vesicle secretion of the rat injure the spermatozoa of the guinea-pig. The possible importance of these discoveries in connection with internal secretions of the so-called prostatic gland, and the possible significance of this again in cases of enlargement of the prostate in elderly men, are obvious, and a wide field for research seems to be opening up in this connection.

